# Spatial Correlations in Attribute Communities

**DOI:** 10.1371/journal.pone.0037507

**Published:** 2012-05-29

**Authors:** Federica Cerina, Vincenzo De Leo, Marc Barthelemy, Alessandro Chessa

**Affiliations:** 1 Department of Physics, University of Cagliari, Cagliari, Italy; 2 Linkalab, Complex Systems Computational Laboratory, Cagliari, Italy; 3 CRS4 Bioinformatica, Pula, Italy; 4 Institut de Physique Théorique (IPhT), CEA, CNRS-URA 2306, Gif-sur-Yvette, France; 5 Institute for Complex Systems, CNR UOS Department of Physics, University of Rome Sapienza, Rome, Italy; Universitat Rovira i Virgili, Spain

## Abstract

Community detection is an important tool for exploring and classifying the properties of large complex networks and should be of great help for spatial networks. Indeed, in addition to their location, nodes in spatial networks can have attributes such as the language for individuals, or any other socio-economical feature that we would like to identify in communities. We discuss in this paper a crucial aspect which was not considered in previous studies which is the possible existence of correlations between space and attributes. Introducing a simple toy model in which both space and node attributes are considered, we discuss the effect of space-attribute correlations on the results of various community detection methods proposed for spatial networks in this paper and in previous studies. When space is irrelevant, our model is equivalent to the stochastic block model which has been shown to display a detectability-non detectability transition. In the regime where space dominates the link formation process, most methods can fail to recover the communities, an effect which is particularly marked when space-attributes correlations are strong. In this latter case, community detection methods which remove the spatial component of the network can miss a large part of the community structure and can lead to incorrect results.

## Introduction

Many networks are embedded in real space and there is a cost associated to the length of links. Examples of such spatial networks can be found in infrastructures such as power grids, distribution and logistic networks, transportation and mobility networks, and also in computer science or biology with the Internet and neuronal networks respectively (see for example the review [1]). Spatial constraints are so important in these networks that one can expect a non-trivial spatial organization as shown in various examples [2]–[10].

In spatial networks, each node is described by its coordinates (usually in a 2d space) but has in general other attributes. For individuals, it can be any cultural or socio-economical parameter. For infrastructure networks such as power grids, it can be the voltage at the electric substations. In general, this attribute depends on space and the resulting network displays entangled layers of parameters. An important goal in the analysis of these networks is to disentangle these different levels and to extract some mesoscopic information from the spatial network structure. If one is interested in studying effects beyond space [5], one should have a straightforward way to ‘subtract’ it from the network, or in other words, to disentangle space and the other attributes.

A natural tool for such a task is community detection which was used for the characterization at a mesoscopic scale of the properties of complex networks (see [11] for a review). A (real-world) community can be naturally defined as a group of network elements having the same attribute value such as language or age for social networks, or the internet domain name for web pages. At a more quantitative level, a community can be thought as a set of nodes more densely linked with each other than with the rest of the network [12]. Community detection procedures consist in finding these groups of nodes in the network. Various methods were proposed so far and we refer the interested reader to the review [11]. In particular, the Newman-Girvan method [13] which relies on the optimization of a quantity called modularity is frequently used and despite its intrinsic limits shown in [14], it possesses the advantage of being simple and relatively easy to implement.

Community detection can have several purposes in spatial networks [2], [4], [15], [16], but probably the main one is to disentangle these various aspects, including spatial correlations of any type. In most cases [2], [4] communities are determined by the geography only, which results from the simple fact that the most important flows are among nodes in the same geographical regions. In this sense, community detection in spatial networks offers a visual representation of large exchange zones. This even suggests that community detection might be an important tool in geography and in the determination of new administrative or economical boundaries [8].

In the general case, for a given network we don’t know to what extent the existence of a link between a pair of nodes is due to a specific factor or to space only. The link could exist because of a strong attribute affinity between the nodes, or in the other extreme case, because they are close neighbors. In general, one could expect a combination of these two effects. If we are interested in recovering communities defined by an attribute (such as language for example) from the network structure, we then have to consider various assumptions such as the correlation between link formation, attribute values and space. In order to understand the effect of the underlying correlations, we can consider two extreme cases. When the links are purely spatial and independent from the attributes, if we remove the spatial component, we will observe random communities (obtained for a random graph) which contain a random number of nodes with random attributes. In this situation, community detection is unapplicable and there is no way to recover attribute communities from the network structure. The other extreme case is when the formation of a link depends on the attributes only. In this case, space is irrelevant and any standard community detection method should give sensible results, ie. communities made of nodes with the same attribute.

The important problem of interest here is thus the intermediate case when the probability to have a link depends both on attributes and on space. In this case we have to eliminate spatial effects in order to recover the attribute structure. An important point in the discussion is then the existence of correlation between space and attributes. The nature and existence of these correlations will govern the way we will have to do community detection. In this paper, we construct a simple artificial network model allowing us to investigate the effect of these correlations on the results of the community detection procedure. We will test various methods on this toy model.

**Figure 1:**
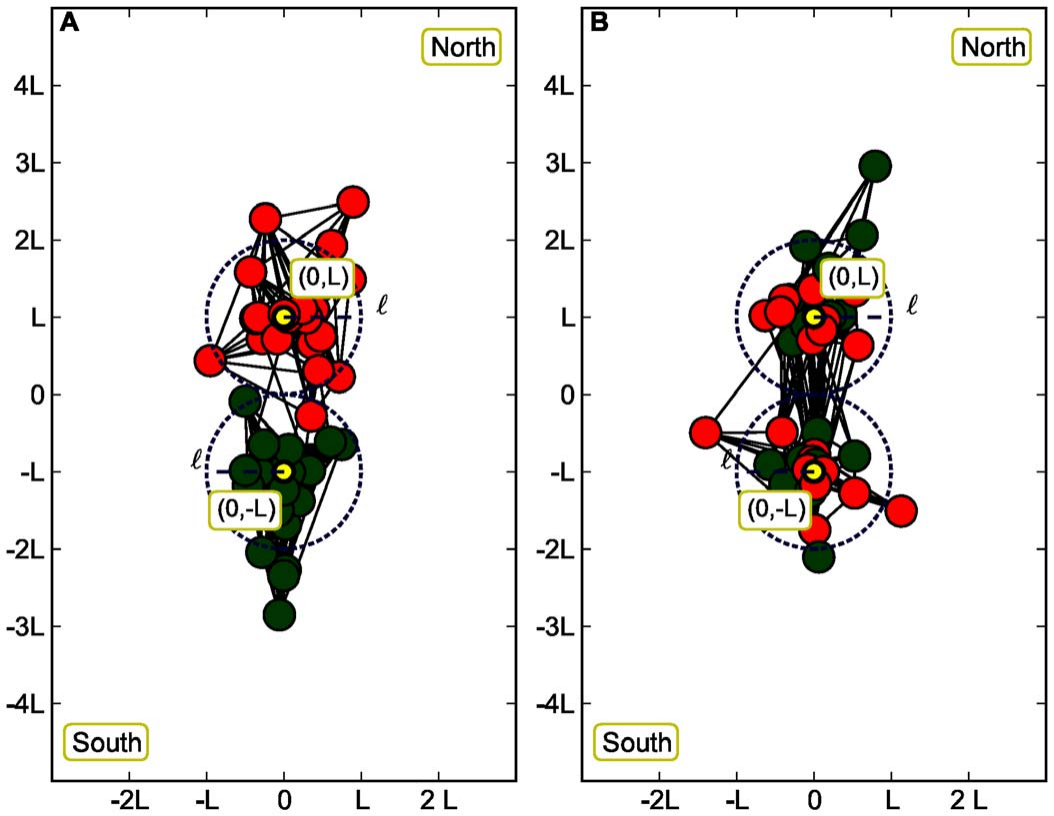
The two spatial communities North and South are well separated having their average size. 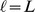
. In the A panel we present the case 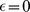 where there is a perfect correlation between the space and the attributes (green and red colors). In the B panel, the uncorrelated case 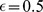 is presented where the attribute colors are randomly distributed between the two segregated spatial communities (for the sake of clarity, only 40 out of the 100 nodes used in our simulations are shown here, and 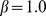).

## Materials and Methods

In order to test these ideas and how community detection acts on spatial networks, we define a simple model of spatial networks with attributes. The attributes could be anything and we will restrict - without loss of generality - to the simple binary case where the attributes can have two possible values at each node. We will introduce a simple model where nodes and their attributes are randomly distributed in space. In general, according to the various parameters of the model, the attributes can be delocalized in space or, on the contrary, be localized in some well-defined region. In some cases, some attribute community could emerge in space, but our target community structure will always be the partition of the network in the two subgraphs composed of nodes with the same attribute and we will test how various methods can recover these two communities. In this respect the main focus of our work will be the disentanglement of the sole attribute network features beyond the spatial node arrangements.

**Figure 2:**
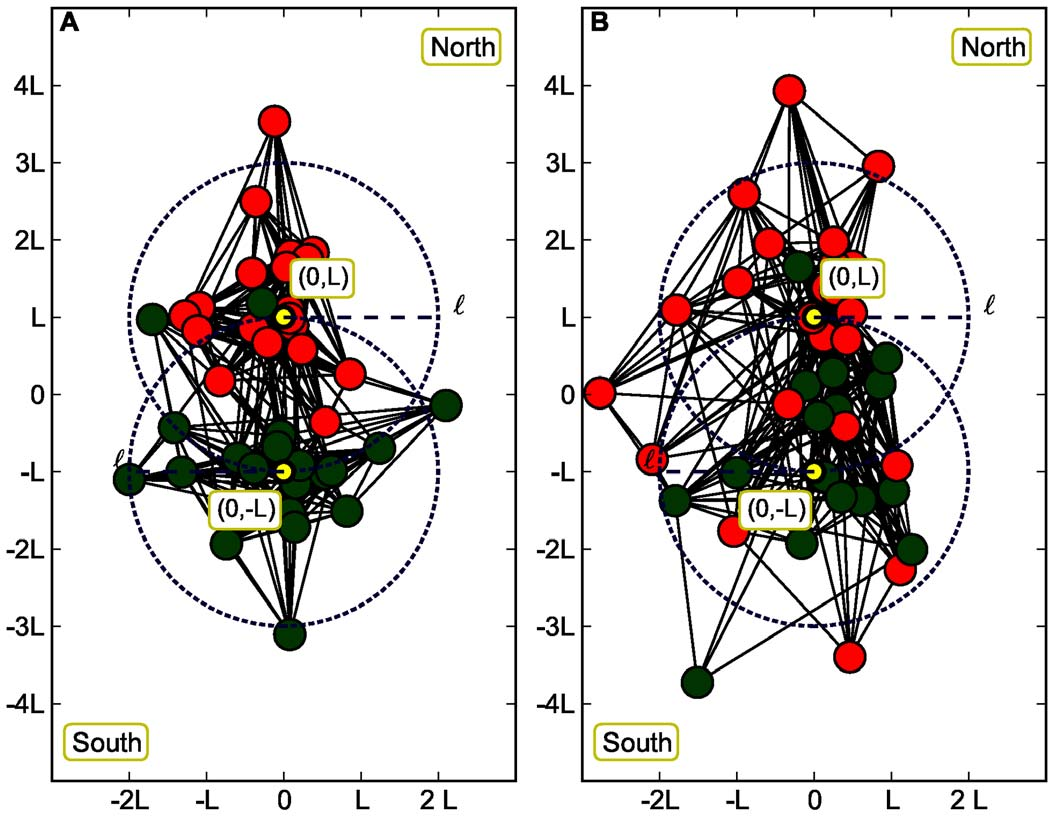
The two communities North and South are mixing up each other with their average size 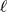 approaching the value of *L* (in this case 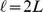). In the A panel, we display the case 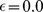. Even if the spatial correlation is fading away the space-attribute correlation is still strong enough to display an attribute community. In the B panel, we show the extreme case 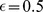 where the attributes are not correlated with space. In this case spatial mixing destroys the attribute community structure (for the sake of clarity, only 40 out of the 100 nodes used in our simulations are shown here, and 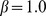).

**Table 1:** Behaviour of the model in the regimes 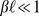 and 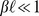.

 Spatial correlation <$>\scale 100%\raster="rg1"\<$>	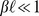 : Space is the governing factor	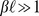 : The spatial component of the links is irrelevant
Spatially correlated: ( 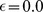 )	• Links are between neighboring nodes but spatialcommunities correspond to the attribute ones.• Any regular community detection will work.	• Links are between nodes with the same attribute.• Any community detection method should work.
Spatially uncorrelated: ( 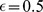 )	• Links are between neighboring nodes but theattributes are anywhere in space.• It is necessary to ‘remove’ space in orderto uncover the attribute communities.	• Links are between nodes with the same attribute.• Any community detection method should work.

The table gives an account of the behaviour of the model in the regimes 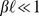 and 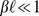 both in the correlated (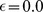) and uncorrelated (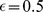) case.

We construct the test (benchmark) network defining the vertex and edge properties in the following way.

### Vertex Properties

1. We generate points/nodes in the 2*d* space (*x*−*z*) in two spatial communities, say the North and the South, around the two centers 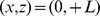 and 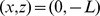 (see Fig. 1). A simple way to do that is to generate points *i* around the two centers according to the probability.
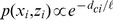
(1)where 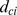 is the euclidean distance between one of the centers *c* and the node *i* of coordinates 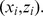


2. We assign an attribute 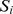 to each node *i*. In the following we will focus on the simplest case where this attribute can take only two values 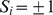 (which in this paper are the red and green colors). A simple way to control correlations between attribute and space is to choose 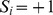 with probability *q* for 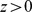 and 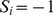 with probability 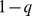. In order to tune the various cases we introduce the parameter 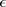, with 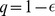, that determines the mixing between space and attributes, ranging from 0.0 to 0.5. In the case 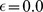 space and attributes are strongly correlated, while for 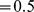 space and attribute are totally uncorrelated.

So the relevant parameters for the generation of network nodes are 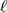 and 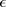.

### Edge Properties

3. We then construct the network: for each pair of nodes, we create a link between nodes *i* and *j* with probability 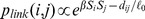 where 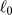 plays the role of the typical size of the spatial community (and where 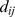 is the euclidian distance between *i* and *j*). It is worth observing that the parameter 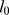 is the typical length of links when space dominates while 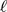 is the typical spatial size of the northern and southern communities. Here the relevant edge parameters are 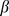 and 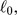 but in order to simplify the model and to focus on the efficiency of community detection methods, we choose 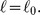 This choice implies that when space dominates the link formation, the links cannot be much larger than the community size. In this case, the only spatial relevant parameter will be 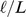 and we can fix *L* to be equal to 1.0 so that the spatial variability will be governed by 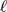. We can rewrite the probability 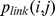 as
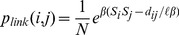
(2)where 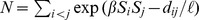 is the normalization constant. As in the Erdos-Renyi random graph, the number of edges is a random variable with small fluctuations around its average. The number of nodes is thus fixed in each network but not the number of edges or the average degree, and this implies that we will have to average our observables over different realizations of the network.

When 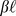 is large, links are essentially between nodes with the same attribute (irrespective of their distance) and if 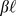 is small then space is the governing factor and links are essentially between neighboring nodes.

In this way the probability associated to a link depends on both space and attribute, and the correlation between attributed and space can be controlled. If the attribute is the same between two nodes the probability to have a link will be reinforced, otherwise it will be weakened, the interplay being controlled by the parameter 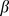. Concerning the spatial factor, the closer the nodes and the larger the probability associated to this link.

The generation of attributes is an important point. We have two values of the attribute only so that we need to generate attributes for only half (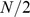) of the nodes. So in the following we will study the specific case of an attribute community structure of equal size communities: half of the nodes has attribute 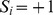 and the other half has 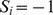. We will investigate here two extreme situations:

- Attributes and space uncorrelated: this case is recovered by choosing 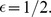

- Attributes and space are strongly correlated. For this, we choose 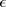 small. In this case, the spatial communities are also attribute communities.

Furthermore we can distinguish two different spatial arrangements for the northern and southern communities. The first case corresponds to a situation where the two communities are well separated with their average size 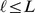 and the spatial effects dominate the community structure (see Fig. 1). The second situation corresponds to a larger value of the average community size 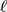 where the two communities start mixing up while 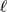 approaches *L* (see Fig. 2).

There are many proposal in the literature for networks benchmarking (see for example [17]), but this is -up to our knowledge- the first one which takes into account the correlation between space and node attributes.

The interplay between space and attributes can lead to various situations that need to be understood within the framework of community detection. Indeed we have two main regimes 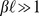 and 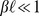 (see also Table 1):

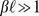
. In this case, the spatial component of the links becomes irrelevant (see Eq. 2) and for a given value of 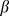 the community structure due to the node attributes will emerge, independently from the correlation between space and attributes. In this regime any community detection method should work.

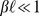
. Here we have two subcases depending con the correlation between space and attributes:
  - (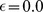) Space and attributes are correlated: any regular community detection will work and moreover if you carefully remove the spatial effect the attribute community structure will be recovered.
  - (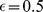) Space and attributes are uncorrelated: in this case the links are between neighboring nodes but the attributes are anywhere in space. Standard community detection methods won’t work and it is then necessary to ‘remove’ space in order to uncover the attribute communities.

**Figure 3:**
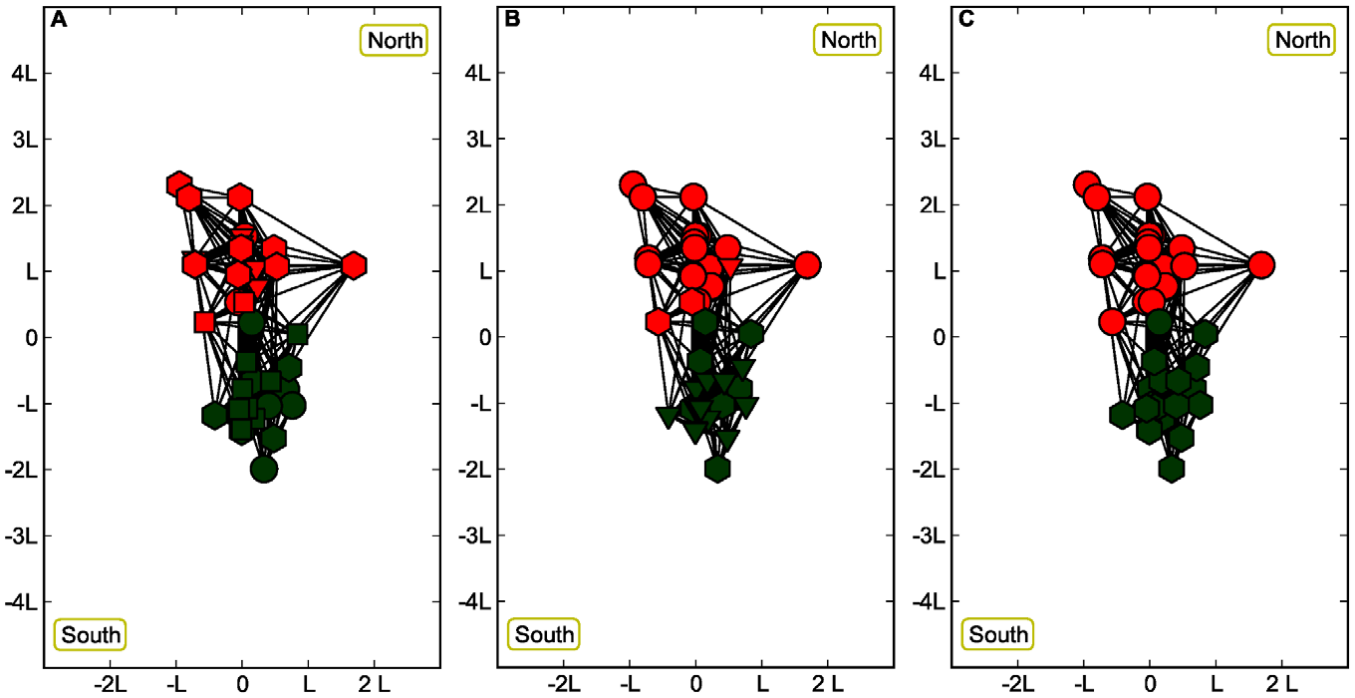
Three spatial network configurations are presented for the constant value 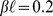 and the correlated case 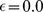 with 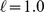 and 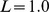. The color (red and green) are the attributes, while the geometrical shapes represent the community memberships found with the various community detection procedure discussed in this paper. In the A panel, we present the case 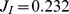, obtained with the Data method. Due to the low 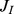 value four communities are present (instead of the two associated with the attributes in red and green colors) and they are also mixed up between the south and the north spatial regions. In the B panel we show the 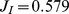 case obtained with the Spatial method. Three communities are present and in the northern part there is a prevalence of circles while in the southern of triangles. The C panel displays the case 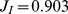 obtained with the Newman-Girvan formulation and the attribute community structure is almost completely recovered.

The general assumption of our model is to what extent it is possible to detect communities even if there is a spatial influence. Without space the initial situation is clear: we have two communities by construction and the probability of two nodes to be connected is related to the attribute similarities. Nodes with S = +1 tend mainly to connect to each other and the same for the S = −1 nodes. If we then put nodes in space and enhance the connection probability due to the proximity of nodes, it is not clear if a regular community detection method is able to detect the original two communities structure. We thus see that correlations between space and attributes can be misleading and any community detection method for spatial networks should take into account this problem. There are now many community detection methods [11] and in the following we will use modularity optimization introduced by Newman and Girvan [13]. This method suffers from various problems, the most important being the existence of a resolution limit [14] which prevent it to detect smaller modules, but it is simple enough to implement. In addition, our point here is to understand the effect of space-attributes correlations on community detection and not to compare various methods. In the following we will thus essentially probe the Newman-Girvan method and variants proposed here and in [5] for cases where the space and attribute have different degrees of correlation.

The modularity function which needs to be optimized is defined as [13]:
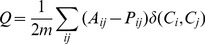
(3)where the sum is over all the node pairs, *A* is the adjacency matrix, *m* is the total number of edges and 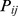 is the expected number of edges between the vertices *i* e *j* for a given null model. The 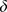 function will result in a null contribution for couples of vertices not belonging to the same community (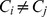). For an unweighted network, one can choose 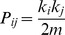 which amounts to take as a null model a random network with the same degree sequence as the original network. In order to introduce explicitly space, the idea is to change the null model defined by 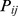 and to compare the actual network with this null model. Recently, such a proposal was made in [5] where the quantity 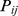 is directly obtained from the data describing the network. More precisely, Expert et al. [5] used the following form
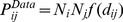
(4)where 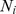 is related to the importance of the node i (such as the population for example). This form is reminiscent of the gravitional model for traffic flows (see for example [18]) where flows are proportional to the product of populations and decrease with distance. In [5], the authors proposed to estimate the unknown function f directly from the empirical data by
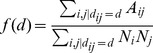
(5)which can be seen as the probability to have two nodes connected at a distance d. Note that there is a binning procedure hidden in Eq. (5). The usual way to proceed in these cases consists in introducing a discretization of the space in bins that capture classes of distances. Following [5], we performed a binning of distances selecting the best value for the number of bins after a detailed stability study of the distributions obtained from the data.

**Figure 4:**
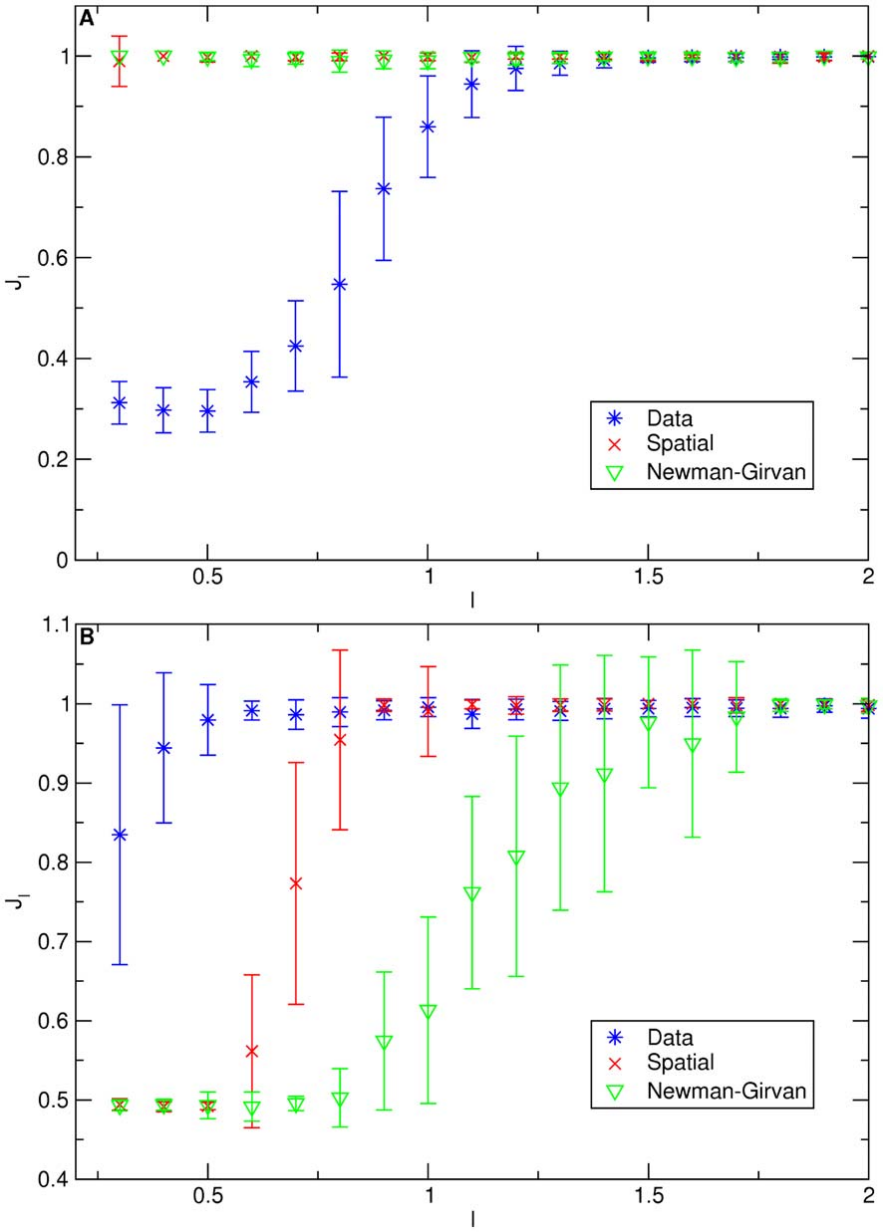
The community structure obtained for various values of 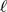 with fixed 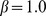. Each point represents the average Jaccard index for 100 network community detection and the error bar is its standard deviation. The correlated case 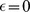 is shown on the A panel, and on the B panel we show the uncorrelated case 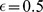. In A for the regime 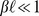 both the Newman-Girvan and the ‘Spatial’ method formulations give the right attribute community structure corresponding to the Jaccard index 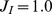. For the regime 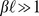 all the three formulations work well since the links due to the attribute similarity are strong enough to preserve the community structure irrespectively from the node’s location. In the uncorrelated case (B panel), the Data based formulation performs better respect to the Spatial formulation, since it extracts correctly the spatial information, directly from the data. In any case both spatial methods reach the right attribute community structure at almost the same value for 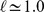. The Newman-Girvan standard formulation instead fails to detect the correct result up to values of 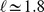. Note that in the x-axis we considered only values equal or above 0.3 since we verified that below this value the model generates disconnected networks.

Expert et al. [5] applied this method to the specific case of the phone network in Belgium, and try to reconstruct linguistic communities (Flemish and French) beyond individuals spatial location. This choice is probably the best one if there are no correlations between the attribute under study (in their case the linguistic membership of the people calling each other) and space. In this specific case, extracting the node spatial dependencies from the actual link distribution present in the network data is the most effective way to subtract the spatial component. Otherwise if there are any correlations between space and node attributes, the data contain in an unknown proportion the two informations (space and attribute) and their method needs to be reformulated. One possible way to do this is to explicitly guess a spatial dependency of the link distribution and to put it as an independent factor in the optimization function definition. In order to be able to deal with the correlated case and to remove spatial effect only, we thus propose the following explicit function of space for 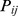


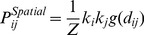
(6)where *Z* is the normalization constant, 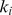 the degree of the node *i*, 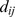 the euclidean distance between node *i* and node *j*. The function 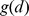 is a decreasing function of distance and its role is to remove the spatial effect. A simple choice is
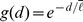
(7)where 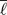 is the average distance between nodes in the network. Of course 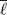 is a rough approximation of the real 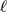 value, but we will see in the following that it is enough to capture the essence of the spatial signature of the network.

We now need a method to compare the community structure obtained with the modularity optimization and the expected one for the attribute membership. Many proposals have been introduced [19]–[21], and we decided to use here the *Jaccard Index* [22], [23]. This index is an extension of the Rand index [24], and is considered to be one of the most robust measure for the clustering and classification assessment of graphs [25]. If *C* is the partition to be evaluated and 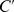 the reference one the definition is as follows
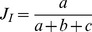
(8)where *a* is the number of vertices pairs that are in the same community for both *C* and 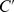, *b* is the number of pairs that are in different communities in *C* but in the same one in 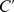 and finally *c* is the number of vertices pairs that are in the same community in *C* but not in 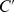 (or conversely). This quantity 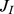 is in the interval [0,1] and the closer to one, the better the agreement between the two partitions. For 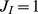 there is a perfect match between the two community structures. In our case, it would mean that the attribute communities are exactly detected. For values of 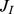 less than 1 the discrepancy can depend both on the size of the partitions in the community structure and/or the number of them and in this respect the *Jaccard Index* is a good method to compare a very heterogeneous range of community structures.

In order to get a more intuitive picture of the Jaccard index, we show three different cases in Fig. 3 for the same value 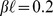 (and in the case 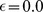, 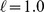 and 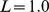) but with different values of 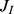. The first case corresponds to a relatively small value 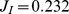 (obtained with the ‘Data’ method of [5], where the binning is done as in their paper, which shows a partition in four communities (instead of the two associated with the attributes in red and green colors). For intermediate values such as 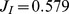 (obtained with our ‘Spatial’ method) the communities reduce to three with a prevalence of circles in the nothern part and triangles in the southern (see B panel in Fig. 3). The last case (obtained with the original Newman-Girvan formulation) corresponds to a value 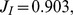 that almost recovers the attribute community structure.

Finally, in order to have a baseline value we also computed the average Jaccard for a completely random partition for 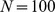 nodes and we obtain the value 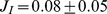.

**Figure 5:**
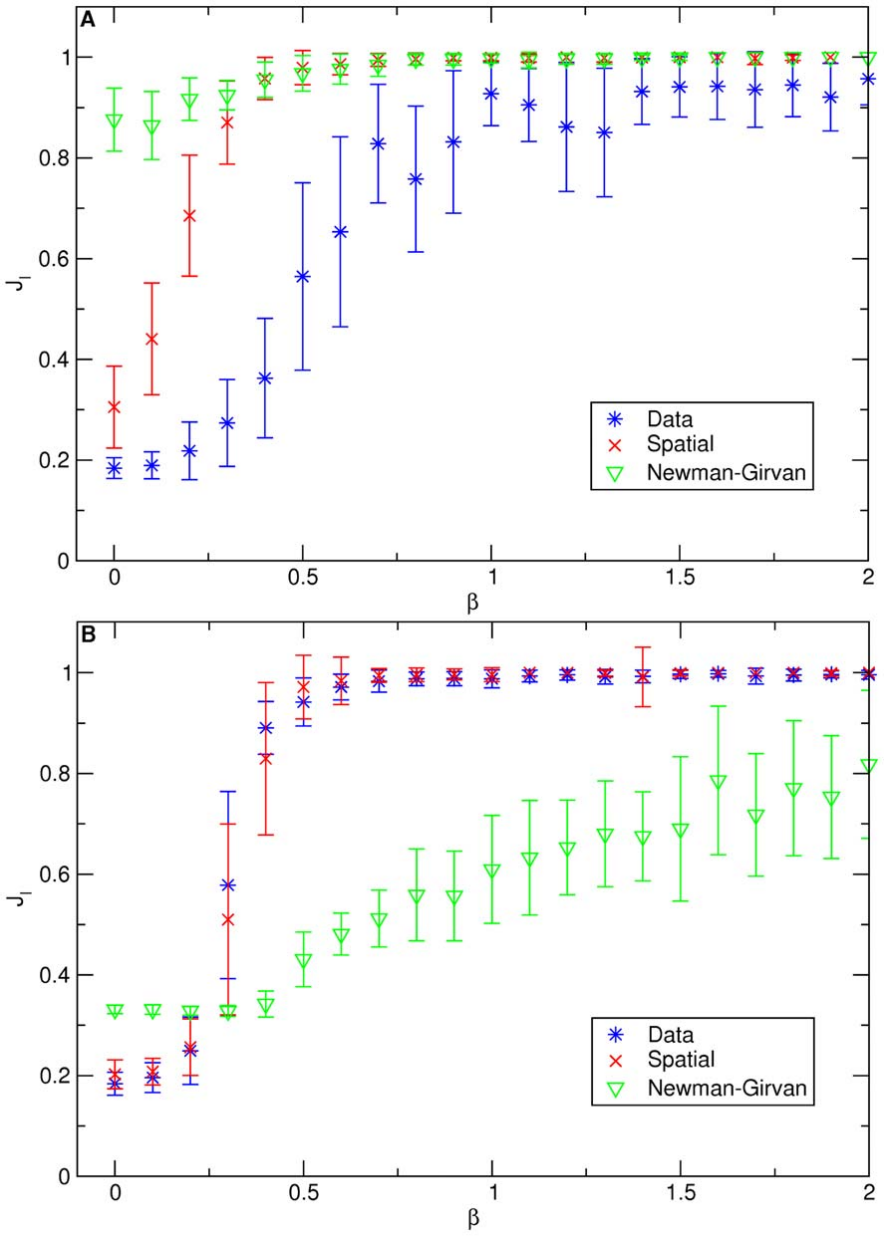
The community structure obtained for various values of 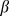 with fixed community size 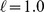. Each point represents the average Jaccard index for 100 network community detection and the error bar is its standard deviation. The correlated case 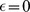 is shown on the A panel, and on the B panel we show the uncorrelated case 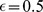. In the uncorrelated case the ‘Data’ method fails in detecting the attribute community structure for all the 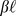 regimes present in the figure, while the other two methods start working at 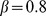. In the uncorrelated case the Newman-Girvan method is not able to detect the attribute community structure, while the spatial methods perform similarly better approaching the correct 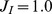 value around 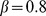.

**Table 2:** Summary of the performances.

 Spatial correlation <$>\scale 100%\raster="rg1"\<$>		Newman-Girvan	Data	Spatial
2*0.0 (correlated)	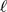	VG	B	VG
	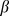	VG	B	G
2* 0.5 (uncorrelated)	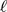	B	VG	G
	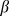	B	G	G

The table summarizes the performances, as can be extracted from Figs 4 and 5, of the three methods (Newman-Girvan, Data and Spatial) in the only non trivial regime 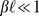, both in the correlated (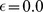) and uncorrelated (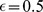) case. Since in the plots we vary both 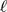 and 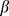, we distinguish here these two cases. In order to be able to compare this results we classified them according to the following criteria: **B**, **G** and **VG** that stand for **Bad**, **Good** and **Very Good**. We assign VG when there is a very good agreement with the target attribute community structure (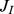 very close to 1), G when the behavior is rapidly approaching the correct result even for low/medium values of the parameters 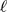 and 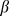, and finally B when it completely fails to recover the right community structure.

## Results

The goal of this spatial community detection is to substract the spatial component and to recover the (two) attribute communities. We thus have three community detection methods: the original Newman-Girvan method, the ‘Data’ method proposed in [5], and our ‘Spatial’ method defined by the null model of Eq. (6) and, in order to understand their limits, we will test them against the benchmark network introduced above.

**Figure 6:**
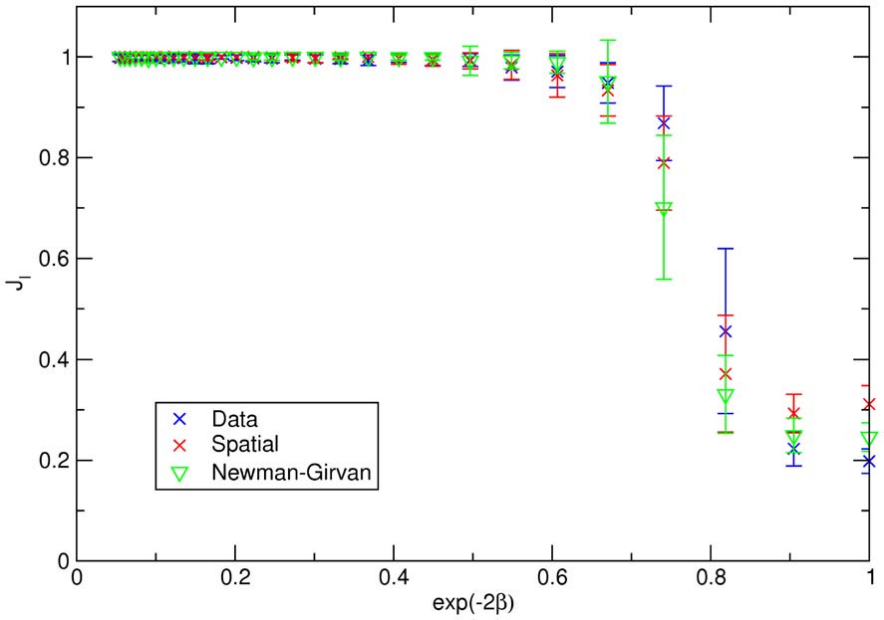
Transition obtained in the case 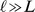 from the detectable to the undetectable community structure regions. This transition was described in [28] for the stochastic block model which corresponds to our model with 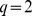 attributes when the effect of space is absent, i.e. 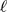 large (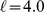 in the actual simulation). The control parameter is then 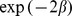 and the Jaccard index is our order parameter. All the three community detection methods discussed in this paper display the same behavior adding evidence to the universality of the transition presented in [28].

**Figure 7:**
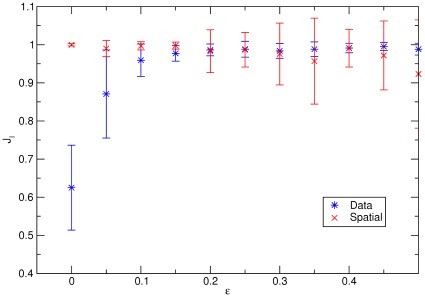
Performances of the Spatial and Data modularity formulations. We show here the case 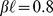 where there is a crossover in the performances around 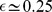. Below this value 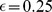 the Spatial method performs better and above the Data method is slightly better.

We will now see how these three different methods perform in the two extreme cases of attribute correlated (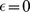) and uncorrelated (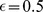) with space, both varying the size of the spatial communities 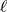 and the attribute linkage strength 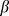. The size of the test network is 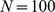 nodes and the number of links depends on the probability previously defined (Eq. 2). We generated 100 network realizations for each set of parameters (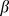, 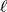,  and 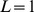). For each point of the simulation curve the error bars are the standard deviation for 100 modularity measures. To optimize the modularity we used the Louvain method [26].

The behavior of the model depends on both parameters 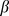 and 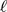 and we will first show the case with fixed attribute strength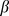. We show on the A panel of figure 4 the correlated case (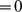) with a fixed 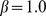.

In this case, for 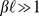, all the three methods work well, as expected and we obtain a perfect match (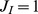) between the community structure resulting from the modularity optimization and the attribute communities. Space is not relevant in this regime and links exist essentially among nodes with the same attribute. For 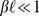 both the Newman-Girvan modularity and the ‘Spatial’ method give the correct result. The latter actually subtract only the spatial dependency while the the ‘Data’ method mixes the space effect with the correlated attribute feature, resulting in a wrong community detection. The ‘Data’ method, for a sufficiently large value of 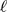 will approach anyway the correct 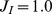 value.

In the uncorrelated case (Fig. 4, B panel) and for a low values of 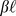, the Newman-Girvan modularity is not able to detect the right attribute communities, since the attribute correlation is not strong enough to group together the nodes of similar type. Instead the other two methods perform better in getting the attribute communities since they are able to correctly eliminate the effect of space and recover the attribute community structure, even for a small attribute correlation. The formulation based on Data performs even better since it eliminates the effect of space almost pointwise, but in any case the correct result of 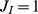 is reached almost at the same value 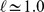 for both spatial methods.

In Figure 5 we show the results for the case of a fixed community size (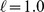) but where we vary the attribute strength 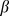. In the A panel the correlated case is presented (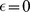). As expected the ‘Data’ method for low values of 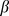 has problems in detecting the attribute community structure and only for high attribute strengths (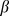) it starts to correctly detect the target communities. In the uncorrelated case, where the space is irrelevant, the standard Newman-Girvan formulation fails, while the two spatial methods performs similarly better (Fig. 5).

In order to summarize these results we show in Table 2 the only relevant regime (b) previously defined, 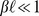 (the (a) regime 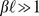 is trivial as we can verify in Figs 4 and 5) for all the parameters of interest (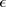, 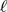 and 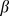) and for the three community detection methods. From this Table, it clearly emerges that the Spatial method is a very good interplay in all situations, while to get the best performances one has to choose the suitable method for any specific case.

We note that the behavior of the error bar sizes in these figures 4, is interesting. For 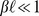 and 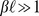, the error in the modularity estimate is relatively small. The error bar -or equivalently the fluctuations of the Jaccard index- are the largest for 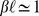. In this region, the community detection methods are thus more sensitive to small fluctuations of the network which implies a peak in the ‘susceptibility’ of the system. This behavior is reminiscent of the phase transition between detectability and non-detectability presented in [27], [28]. Indeed, in figure 6 we show the limiting case of 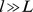 (here we choose numerically 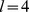 and 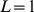) for which the effect of space is irrelevant. In this limit, our model becomes equivalent to the stochastic block model of [28] with 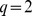 possible values of the attribute. In our case the control parameter (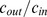 in [28]) is 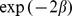, while the order parameter is the Jaccard index. It is clear from Fig. 6 that the same effect is present (see figure 2 in [28]) even if the critical point is shifted due to a different community detection method and another definition of the order parameter. Moreover, respect to the result in [28], in the undetactable regime (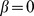), the value of the order parameter is not zero. As mentioned above, for a completely random partition the 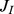 is 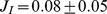. We observe that in our case we are a little bit above because it is known that even for a random network the modularity can be positive [29] and in this way the maximization of the modularity extracts a subset of the ensemble of all the possible partitions that increases the average modularity and consequently the average Jaccard index.

We thus recover the results of [28] and in addition our result seems to point to the existence of a spatial phase transition actually independent of the community detection method used.

Finally, we checked the performances of the Data and Spatial formulations looking at the 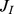 values when varying the  parameter for a fixed 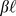 value (see Fig. 7). For each value of  an higher 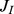 value signals a better behavior since it is closer to the maximum value 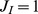. We choose first the value 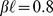 (we also tested 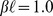 which gives similar results). There is a crossover in the performances around 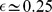. Below this value, the Spatial method performs better while above that point the Data method does slightly better. This result thus shows that there can be a non-negligible range of correlations (measured here by 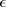) for which the spatial community detection results can be incorrect.

## Discussion

In this paper we propose a simple model which allows us to test community detection on spatial networks. Our model generates simple graphs that mix both geographical properties and attributes. In the literature many other spatial network models have been introduced for which nodes are connected each other through a certain spatial rule. Examples range from the growth of street networks to the evolution of the territorial infrastructural networks (see [1] for an extensive list of this kind of models). Moreover a whole class of models that study node properties and their aggregation has recently been introduced and one of the most important of them is the stochastic block model in which a combination of various kind of node attributes are present. The novelty of our approach is to study at the same time these various aspects (geography and attributes), and, up to our knowledge, our model is the first one that considers simultaneously the two factors, space and attributes, in the context of community detection.

In particular, we explicitly show that the existence of correlations between attributes and space drastically affects the result of community detection. The results presented in this study show that community detection in spatial networks should be taken with great care, and that including space in community detection methods could lead to results difficult to interpret. We show that for weak correlations, most community detection methods work, but that for stronger correlation community detection methods which remove the spatial component of the network can lead to incorrect results. It is thus important to have some information on the correlations between space and attributes in order to assess the validity of the results of community detection methods. In practical applications however, these attributes-space correlations are generally not known and this calls for the need of new approaches, for example such as community detection methods including in some tunable form the existence of such correlations.

## References

[1] Barthelemy M (2011). Spatial Networks.. Physics Reports.

[2] Guimerá R, Mossa S, Turtschi A, Amaral LAN (2005). The worldwide air transportation network: Anomalous centrality, community structure, and cities’ global roles.. Proceedings of the National Academy of Sciences.

[3] Barrat A, Barthelemy M, Vespignani A (2005). The effects of spatial constraints on the evolution of weighted complex networks..

[4] Kaluza P, Kolzsch A, Gastner MT, Blasius B (2010). The complex network of global cargo ship movements.. Journal of the Royal Society Interface the Royal Society.

[5] Expert P, Evans TS, Blondel VD, Lambiotte R (2011). Uncovering space-independent communities in spatial networks.. Proceedings of the National Academy of Sciences of the United States of America.

[6] Daraganova G, Pattison P, Koskinen J, Mitchell B, Bill A (2011). Networks and geography: Modelling community network structures as the outcome of both spatial and network processes..

[7] Chavez M, Valencia M, Latora V, Martinerie J (2010). Complex networks: new trends for the analysis of brain connectivity.

[8] De Montis A, Caschili S, Chessa A (2011). Commuter networks and community detection: a method for planning sub regional areas.. eprint arXiv:.

[9] Calabrese F, Smoreda Z, Blondel VD, Ratti C (2011). Interplay between telecommunications and face-to-face interactions: A study using mobile phone data.. PLoS ONE.

[10] Grady D, Brune R, Thiemann C, Theis F, Brockmann D (2011). Modularity maximization and tree clustering: Novel ways to determine effective geographic borders.. eprint.

[11] Fortunato S (2010). Community detection in graphs.. Physics Reports.

[12] Porter MA, Onnela JP, Mucha PJ (2009). Communities in networks.. Notices of the American Mathematical Society.

[13] Newman MEJ, Girvan M (2004). Finding and evaluating community structure in networks.. Physical Review E - Statistical, Nonlinear and Soft Matter Physics.

[14] Fortunato S, Barthelemy M (2006). Resolution limit in community detection.. physics0607100.

[15] Hu D, Ronhovde P, Nussinov Z (2011). A replica inference approach to unsupervised and multi-scale image segmentation..

[16] Gregory S (2011). Ordered community structure in networks.. Physica A.

[17] Lancichinetti A, Fortunato S (2009). Community detection algorithms: a comparative analysis.. Physical Review E - Statistical, Nonlinear and Soft Matter Physics.

[18] Erlander S, Stewart NF (1990). The gravity model in transportation analysis: theory and extensions/Sven Erlander and Neil F. Stewart..

[19] Danon L, Duch J, Arenas A, Díaz-Guilera A (2005). Comparing community structure identification.. Journal of Statistical Mechanics: Theory and Experiment.

[20] Campello RJGB (2007). A fuzzy extension of the Rand index and other related indexes for clustering and classification assessment.. Pattern Recognition Letters.

[21] Karrer B, Levina E, Newman MEJ (2007). Robustness of community structure in networks.. Physical Review E.

[22] Jain AK, Dubes RC (1988). http://portal.acm.org/citation.cfm?id=42779.

[23] Halkidi M, Batistakis Y, Vazirgiannis M (2001). On clustering validation techniques.. Journal of Intelligent Information Systems.

[24] RandW (1971). Objective criteria for the evaluation of clustering methods.. Journal of the American Statistical Association.

[25] Denoeud L, Garreta H, Gu A (2006). Comparison of distance indices between partitions..

[26] Blondel VD, Guillaume JL, Lambiotte R, Lefebvre E (2008). Fast unfolding of communities in large networks..

[27] Hu D, Ronhovde P, Nussinov Z (2011). Phase transitions in random potts systems and the community detection problem: spin-glass type and dynamic perspectives.. Philosophical Magazine.

[28] Decelle A, Krzakala F, Moore C, Zdeborová L (2011). Phase transition in the detection of modules in sparse networks.. Physical Review Letters.

[29] Guimerà R, Sales-Pardo M, Amaral LAN (2004). Modularity from uctuations in random graphs and complex networks.. Phys Rev E.

